# First person – Zhaoyang Liu

**DOI:** 10.1242/dmm.043612

**Published:** 2019-12-17

## Abstract

First Person is a series of interviews with the first authors of a selection of papers published in Disease Models & Mechanisms, helping early-career researchers promote themselves alongside their papers. Zhaoyang Liu is first author on ‘Regulation of terminal hypertrophic chondrocyte differentiation in *Prmt5* mutant mice modeling infantile idiopathic scoliosis’, published in DMM. Zhaoyang is a postdoctoral fellow in the lab of Ryan Gray at The University of Texas at Austin, Austin, TX, USA, investigating mechanisms of cartilage development and musculoskeletal-related diseases.


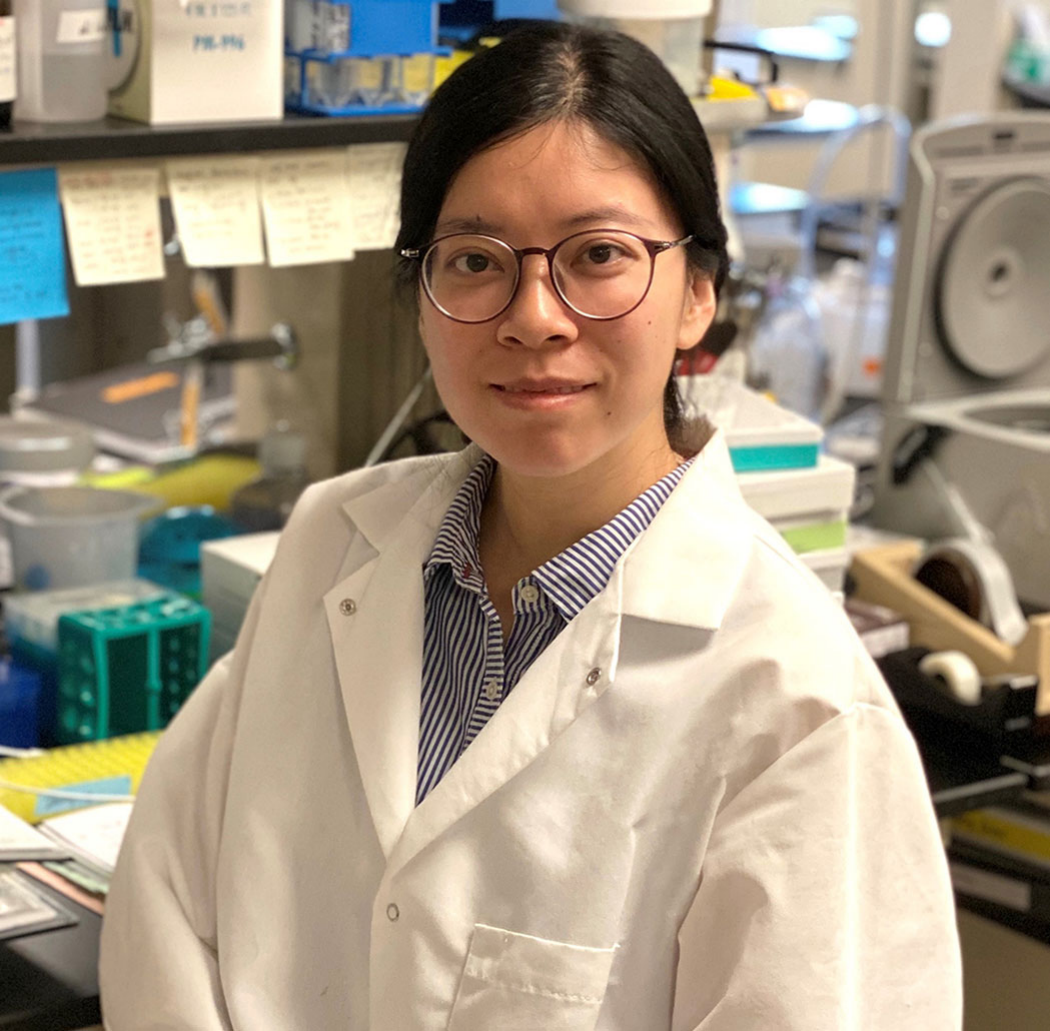


**Zhaoyang Liu**

**How would you explain the main findings of your paper to non-scientific family and friends?**

Idiopathic scoliosis (IS) is an enigmatic disorder characterized by progressive curvature of the spine in otherwise healthy children, and is the most common type of musculoskeletal defect affecting children worldwide. The genetic causes of the disorder, particularly for infantile-onset IS, are not well defined. We set out to determine the role of the PRMT5 protein specifically in cartilaginous tissues in mouse using conditional genetics, which specifically remove gene function in a tissue of interest. We found that PRMT5 conditional mutant mice developed spine curvatures at 10 days of age and had reduced bone formation in the vertebral bodies and ribs, which recapitulates features of infantile IS. We further demonstrated that loss of *Prmt5* in cartilaginous tissues affected key pathways and signaling important for chondrocyte differentiation and bone formation. Our study suggests that dysfunction of PRMT5 leading to impaired bone formation of the axial skeleton is a potential cause of infantile IS, and provides a useful animal model for future discovery of novel therapies for IS.

**What are the potential implications of these results for your field of research?**

Little is known about the cause of IS, which is largely due to a lack of valid animal models. In this study, we generated a conditional genetic mouse model that mimics human infantile IS, and links dysfunction of pathways and signaling important for chondrocyte differentiation and bone formation to infantile IS. We postulate a model where the onset of scoliosis is induced by asymmetric mechanical loading of the spine, as a result of subtle defects in bone formation during perinatal development. In light of these results, high-resolution, low-dose X-ray imaging and analysis of bone quality (bone mineral density) coupled with targeted sequence analysis of the *PRMT5* locus in infantile IS patients is warranted.

**What are the main advantages and drawbacks of the model system you have used as it relates to the disease you are investigating?**

The main advantage of using the mouse model for our study is that it recapitulates the phenotype of infantile IS in humans. Distinct from congenital scoliosis, which is characterized by a failure of segmentation, formation or dysplasia in one or more vertebral units, IS patients do not display overt vertebral malformations. Our mouse model shows no spinal deformity during embryonic development, but rapidly develops scoliosis at neonatal stage, coupled with loss of bone formation in the ribcage and the spine. One main drawback of our mouse model is that these mutant mice are perinatal lethal, potentially due to respiratory distress caused by insufficient chest wall stiffness, which prevents us from further investigating the nature history of this disease.

“I was very excited when we found a novel regulator that plays a critical role during the terminal stage of chondrocyte differentiation and crosstalks with multiple master regulators of chondrogenesis.”

**What has surprised you the most while conducting your research?**

I was most surprised by how *Prmt5* seems to precisely regulate cartilage and bone formation, by blocking flux of signaling and pathways important for hypertrophic chondrocyte differentiation. Chondrocyte differentiation has been extensively studied over the past decades and several master regulators have been described. I was very excited when we found a novel regulator that plays a critical role during the terminal stage of chondrocyte differentiation and crosstalks with multiple master regulators of chondrogenesis. Given that *Prmt5* encodes a key epigenetic enzyme for symmetrical demethylation, I look forward to studying the direct molecular targets of this enzyme in cartilaginous tissues in the future.

**Describe what you think is the most significant challenge impacting your research at this time and how will this be addressed over the next 10 years?**

I think in the field of scoliosis research, the most significant challenge is the complexity of the etiology of this disease, and the lack of genetic animal models. The spine is a complex organ that contains various tissues, including cartilage, bone, connective tissues and innervation of the peripheral nervous system. In the next decades, the distinct and synergistic functions of these tissues that contribute to the pathogenesis of scoliosis should be addressed using different animal models, informed by new insights from human genetic studies of IS. These should also benefit from tissue-specific high-resolution transcriptomic and proteomic analyses of animal models of IS in the next 10 years.

**Figure:**
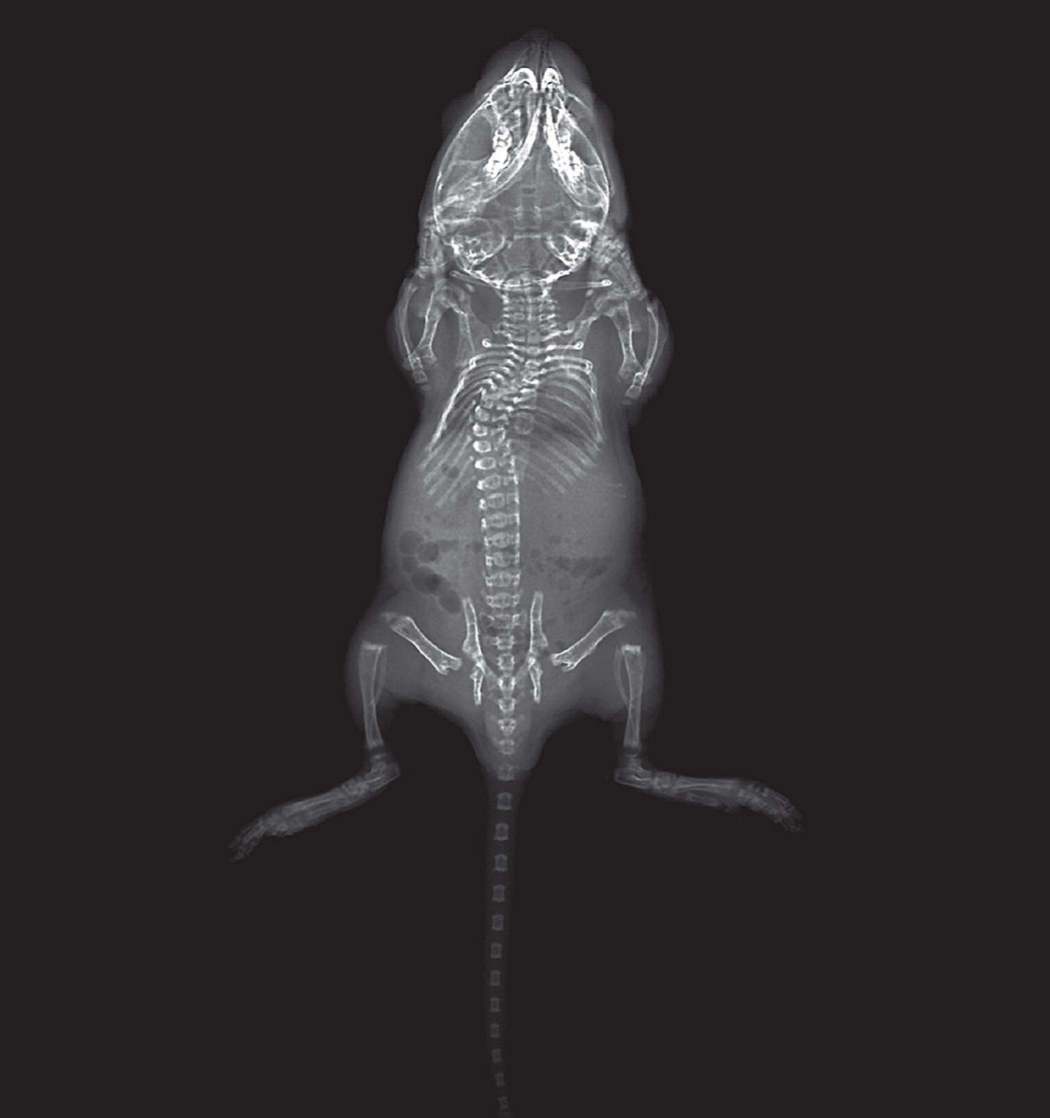
**A 10-day-old *Prmt5* mutant mouse develops scoliosis in the spine.**

**What changes do you think could improve the professional lives of early-career scientists?**

I think changes in academia that can help improve the professional lives of early-career scientists include providing more fellowship and funding opportunities, and it would be very helpful if the ‘post-degree research experience’ eligibility of the applicant can be extended. The early stage of career development is very likely to overlap with many significant life events, so these changes can provide more opportunities and freedom for the grantees to focus on professional development.

**What's next for you?**

I will continue performing translational research in musculoskeletal-related diseases. Besides scoliosis, I'm also involved in several other research projects on musculoskeletal disorders, including intervertebral disc degeneration and osteoarthritis. I'm looking forward to becoming an investigator of musculoskeletal research after my postdoc training.
